# Demystifying the likelihood of reidentification in neuroimaging data: A technical and regulatory analysis

**DOI:** 10.1162/imag_a_00111

**Published:** 2024-03-22

**Authors:** Anita S. Jwa, Oluwasanmi Koyejo, Russell A. Poldrack

**Affiliations:** Department of Psychology, Stanford University, Stanford, CA, United States; Computer Science Department, Stanford University, Stanford, CA, United States

**Keywords:** neuroimaging, data sharing, data privacy, data reidentification, face recognition, regulatory analysis

## Abstract

Sharing research data has been widely promoted in the field of neuroimaging and has enhanced the rigor and reproducibility of neuroimaging studies. Yet the emergence of novel software tools and algorithms, such as face recognition, has raised concerns due to their potential to reidentify defaced neuroimaging data that are thought to have been deidentified. Despite the surge of privacy concerns, however, the risk of reidentification via these tools and algorithms has not yet been examined outside the limited settings for demonstration purposes. There is also a pressing need to carefully analyze regulatory implications of this new reidentification attack because concerns about the anonymity of data are the main reason that researchers think they are legally constrained from sharing their data. This study aims to tackle these gaps through rigorous technical and regulatory analyses. Using a simulation analysis, we first tested the generalizability of the matching accuracies in defaced neuroimaging data reported in a recent face recognition study (Schwarz et al., 2021). The results showed that the real-world likelihood of reidentification in defaced neuroimaging data via face recognition would be substantially lower than that reported in the previous studies. Next, by taking a US jurisdiction as a case study, we analyzed whether the novel reidentification threat posed by face recognition would place defaced neuroimaging data out of compliance under the current regulatory regime. Our analysis suggests that defaced neuroimaging data using existing tools would still meet the regulatory requirements for data deidentification. A brief comparison with the EU’s General Data Protection Regulation (GDPR) was also provided. Then, we examined the implication of NIH’s new Data Management and Sharing Policy on the current practice of neuroimaging data sharing based on the results of our simulation and regulatory analyses. Finally, we discussed future directions of open data sharing in neuroimaging.

## 1 Introduction

The field of neuroimaging has experienced a growing awareness of the importance of sharing research data (Breeze et al., 2012;Choudhury et al., 2014;Mennes et al., 2013;Poldrack & Gorgolewski, 2014;Poline et al., 2012). The scale and scope of shared neuroimaging data have substantially increased during the last decade, and data-sharing initiatives and platforms for neuroimaging data have proliferated across the globe. Some of these initiatives and platforms offer fully open sharing, which means making data publicly available without any restrictions attached, whereas others impose varying levels of restrictions on access to and subsequent use of data (Jwa & Poldrack, 2022a). With the aid of advances in computing and big data analytics, research on pooled, shared data has resulted in hundreds of peer-reviewed publications (Milham et al., 2018) and is expected to further facilitate new scientific discoveries regarding the structure and function of the human brain.

However, there is a critical prerequisite for sharing individual-level human subject neuroimaging data—rigorous privacy and security measures should be in place to protect subjects (Brakewood & Poldrack, 2013). Ethical principles of human subject research oblige researchers to minimize the risk to subjects’ privacy and confidentiality of data, while also maximizing the potential benefits to society from the subjects’ participation (National Commission for the Protection of Human Subjects of Biomedical and Behavioral Research, 1979). In the United States, a multitude of federal and state laws and regulations have stipulated standards and requirements to protect human subjects (U.S. Department of Health and Human Services (HHS), 2003,^2008^). In general, sharing data derived from human subjects for secondary analysis requires redaction of identifiable information, commonly referred to as data deidentification.

Deidentification of neuroimaging data includes careful scrubbing of any potentially identifying fields from the data, such as removing subject names from image file headers or analysis pathnames, or avoiding entry of those identifiers at any point in the data collection process. Another process used to deidentify neuroimaging data is the removal of facial structure from structural images, known as “defacing.” Structural scans in the data contain facial and skull features, and subjects’ faces reconstructed from the data can be used to establish the identity of the subjects, similar to photographic images.

Neuroimaging datasets are often shared with intact facial features in the structural scans when additional privacy measures are in place, such as controlled access to data that is limited to researchers with verified credentials along with a data use agreement that prohibits reidentification of subjects. Yet databases and platforms that publicly share neuroimaging data commonly require defacing to enhance privacy protection. A number of defacing algorithms have been developed that either blur or partially remove facial regions contained in the data (e.g., mask_face (Milchenko & Marcus, 2013), mri_deface (Bischoff-Grethe et al., 2007), pydeface (Gulban et al., n.d.), and fsl_deface (Alfaro-Almagro et al., 2018)). Applying these algorithms, along with the redaction of identifiers from metadata, has been considered sufficient to meet ethical and legal standards for the protection of a subject’s privacy when publicly sharing human neuroimaging data.

Recently, the emergence of advanced face recognition tools has called the effectiveness of defacing as a safeguard against data reidentification into question. Studies have shown that a face recognition algorithm could potentially reidentify neuroimaging data by matching reconstructed faces with subjects’ photos even when the data are defaced (Schwarz et al., 2019,^2021^). Reidentification of the neuroimaging data could further result in unwanted disclosure of additional sensitive information shared alongside the neuroimaging data, such as diagnosis, genetic information, neuropsychiatric measures, or family and personal history. Thus, it has been argued that cutting-edge face recognition tools pose a substantial risk to data privacy and that the current practice of neuroimaging data sharing, especially the open sharing of data, should be reconsidered (D. Eke et al., 2021). Furthermore, in light of increasing prominence of data protection, concerns have been raised that openly sharing defaced neuroimaging data might no longer be compliant with regulatory requirements (D. Eke et al., 2021). More stringent technical and organizational measures that limit access to and secondary analysis of shared data are also called for to address the heightened privacy risk (D. Eke et al, 2021;D. O. Eke et al, 2022;Goering et al., 2021;Ienca et al., 2022;Yuste, 2023).

Despite the surge of privacy concern, however, the likelihood of reidentification in defaced neuroimaging data via these tools and algorithms has not yet been examined outside the limited settings for demonstration (Clunie et al., 2023;Juluru et al., 2020). There is also a pressing need to carefully analyze regulatory implications of this new reidentification attack because concerns about the anonymity of data are a main reason that researchers think they are legally constrained from sharing their data (Paret et al., 2022;Reer et al., 2023). Being more proactive in protecting data privacy would be beneficial given the rapid pace of technological development. But at the same time, it is crucial to critically assess the current state of the reidentification technique and associated privacy risk to avoid an unduly restrictive approach to data sharing, which could fail to maximize the potential benefits of the subjects’ participation as required by ethical principles (National Commission for the Protection of Human Subjects of Biomedical and Behavioral Research, 1979).

The present paper aims to tackle these gaps through a rigorous technical and regulatory analysis and to help researchers navigate the new privacy challenges to neuroimaging data sharing. Through a simulation analysis, we will first test the generalizability of the matching accuracies in defaced neuroimaging data from the recent face recognition studies. Next, by taking a US jurisdiction as a case study, we will analyze how the novel reidentification threat posed by face recognition would affect achieving data deidentification under the current regulatory regime. A brief comparison with the EU’s General Data Protection Regulation (GDPR) will also be provided (European Parliament & Council of the European Union, 2016). Then, we will examine the implication of NIH’s new Data Management and Sharing Policy on the current practice of neuroimaging data sharing based on the results of our analyses. The paper will conclude by discussing future directions of open data sharing in neuroimaging.

## 2 Reidentification of neuroimaging data via face recognition

Neuroimaging encompasses the use of various imaging technologies, such as magnetic resonance imaging (MRI), positron emission tomography (PET), and computed tomography (CT), to study the structure and function of the nervous system in a non-invasive way (Fulham, 2004). Data generated by these technologies have uniquely sensitive characteristics that can be linked to the identity of subjects, particularly the facial features present in structural images. The possibility of reidentifying subjects by reconstructing their face from the images and applying face recognition has long been the major privacy concern around neuroimaging data. For example,Mazura and colleagues (2012)tested a face recognition algorithm (Google Picasa, 2009) on CT-based reconstructed faces and reported a matching rate of 27.5% when compared with subjects’ photographs.

The unprecedented recent development in machine learning and AI has led to a significant improvement in face recognition techniques.Schwarz and colleagues (2019)attempted to reidentify MRI scans (3D FLAIR images) using a state-of-the-art face recognition algorithm (Microsoft’s Azure (Microsoft Corporation, 2019)). For each of the 84 subjects, a 3D computer model of the face was reconstructed from their MR brain images, and 10 2D photograph-like images were created to train an instance of the algorithm to recognize each subject. Then, five facial photographs of each subject taken by the experimenter were used as input to the algorithm to identify a ranked list of the best matches among the set of 84 MRI-based photograph-like face reconstructions. Match confidence scores of 84 face reconstructions for each of the five photos of the subjects were summed to generate the combined ranking of the face reconstructions for individual subjects. For 70 of the 84 participants (83%), the algorithm chose the correct MRI-based face reconstruction as the most likely match for their facial photographs. The correct MRI scan was among the top five choices in the ranked list for 80 of 84 participants (95%).

In their follow-up study with an updated algorithm (2021), Schwarz and colleagues further demonstrated the potential to reidentify subjects even after facial features were removed from structural MR images via defacing (N = 157). The algorithm performed nearly perfectly on original intact FLAIR images (97%), improved from the matching rate of 83% in their 2019 study, but it was also quite effective on defaced images.^1^The commonly used defacing tools prevented the reconstruction of faces in the majority of subjects’ images, but for images in which any parts of a face remain (11%, 13%, and 3% of images defaced with mri_deface, pydeface, and fsl_deface), the face recognition algorithm was highly accurate in matching the reconstructed partial faces with the subjects’ photo. For images defaced with mri_deface and pydeface, the matching rate was 10%; for images defaced with fsl_deface, the matching rate was 3%. On previously defaced images where the facial structure was imputed using a population-average face template^2^(“refaced images”), match rates increased to 33% (mri_deface), 38% (pydeface), and 28% (fsl_deface).

## 3 Generalizability of the likelihood of reidentification via face recognition in neuroimaging data

At first glance, the rather surprising results from the face recognition studies raise serious concerns about the adequacy of defacing for neuroimaging data deidentification. In response to these alarming reports, discussions have ensued on whether the existing defacing tools for neuroimaging data would provide sufficient protection required under the ethical and regulatory standards. However, it is important to note that these results cannot be directly translated into the likelihood of reidentification in real-world situations.

### 3.1 Threat model

Before delving into the examination of the generalizability of reported matching accuracies, it would be important to clarify our threat model—a formal description of the privacy setting. Here, we assume the attacker can access individual information about the full target population (e.g., public images) and the imaging data from a research study. Given a participant from the potential population, the attacker seeks to identify if the individual participated in the study—thus identifying their associated brain image. We do not assume the attacker has computational restrictions.

### 3.2 The issue of the size of the potential target population

The reidentification problem is an example of a multi-class classification problem, wherein each individual represents a separate class (Aly, 2005). In these problems, it is well-known that classification accuracy is necessarily tied to the number of classes being distinguished. All else being equal, it would be more difficult to accurately identify an individual out of a lineup with more distractors versus fewer distractors, with accuracy decreasing in a roughly exponential way with the number of classes (Kay et al., 2008;Slavutsky & Benjamini, 2020;Zheng et al., 2018).

For most real-world reidentification problems, the pool of potential matches is much larger than the size of samples in the previous studies is (N = 157). For example, take a data set collected from adults ages 20–49 at a research center in the Pittsburgh, Pennsylvania, metropolitan area. The pool of potential targets for reidentification in such a study would comprise all individuals in the Pittsburgh metropolitan area who meet the inclusion criteria. According to the 2022 US Census statistics, 36.8% of the population of 2,349,172 falls within this age range, giving a potential pool of roughly 865,000 individuals who could have participated in the study (United States Census Bureau, 2022a,2022b).

The size of the pool can be substantially decreased with additional demographic information commonly shared with neuroimaging data, such as gender, age, or race/ethnicity, but is still expected to be greater than the size of the samples in the previous studies. In the example above, let us assume that the gender of the target subject is known (e.g., female) in addition to the age range (20–49). Then, the size of the population is reduced from 865,000 to roughly 423,000 (18% of the total population in the area) (United States Census Bureau, 2022a). If we can narrow down the age range to, for example, 25-29, it further reduces to roughly 70,000 (3.2% of the total population). Finally, if we also know the race of the subject (e.g., Black), the size of the population becomes around 6,500 (United States Census Bureau, 2022b).

Unfortunately, there are no data sets containing both facial photos and MRI images that are of sufficient size to test the real-world effectiveness of reidentification in such a sample. Instead, here, we demonstrated the way in which classification accuracies are related to base rates through a simulation analysis. We designed a classification problem, which is simpler but comparable to the reidentification problem in previous studies and simulated the performance of the classifier as the population size increases.

### 3.3 Simulation analysis

We generated a population of individuals with 1,000 features each (sampled from a normal distribution) and trained a one-nearest-neighbor classifier to identify the individuals using a Euclidean distance metric. Test data were also generated by adding random noise to each individual; the amount of added noise was calibrated to provide a target level of reidentification performance based on the reported accuracies of Schwarz and colleagues’ study (either 10% or 38%, based on the reidentification levels observed bySchwarz et al. (2021)for the pydeface defacing tool) for a sample size of 157 as in their study.

We then assessed reidentification performance for that level of signal-to-noise as the population size varied from 157 to a size from the example of the Pittsburgh, Pennsylvania, metropolitan area—6,500 (a Black female, age 25–29), 70,000 (a female age, 25–29), 423,000 (a female age, 20–49), and 865,000 (an adult, age 20–49). (https://doi.org/10.5281/zenodo.10815242; the results can also be viewed athttps://github.com/poldrack/reidentification-simulations).

It should be noted that neuroimaging datasets typically contain exact integer ages of the subjects. However, we could not conduct the analysis on the population size of a single age group because the US Census data solely provides 5-year age group population estimates for this area (United States Census Bureau, 2022a,2022b). Instead, we used the ranges of age to demonstrate the effect of having more detailed demographic information—broad age range (20–49) to narrow age range (25–29)—on the size of the target population and, in turn, on the performance of face recognition. If the attacker had information on the number of individuals who are of the same age as a subject in a certain geographic area, that would substantially reduce the size of potential matches and increase the likelihood of reidentification.

**Fig. 1.**
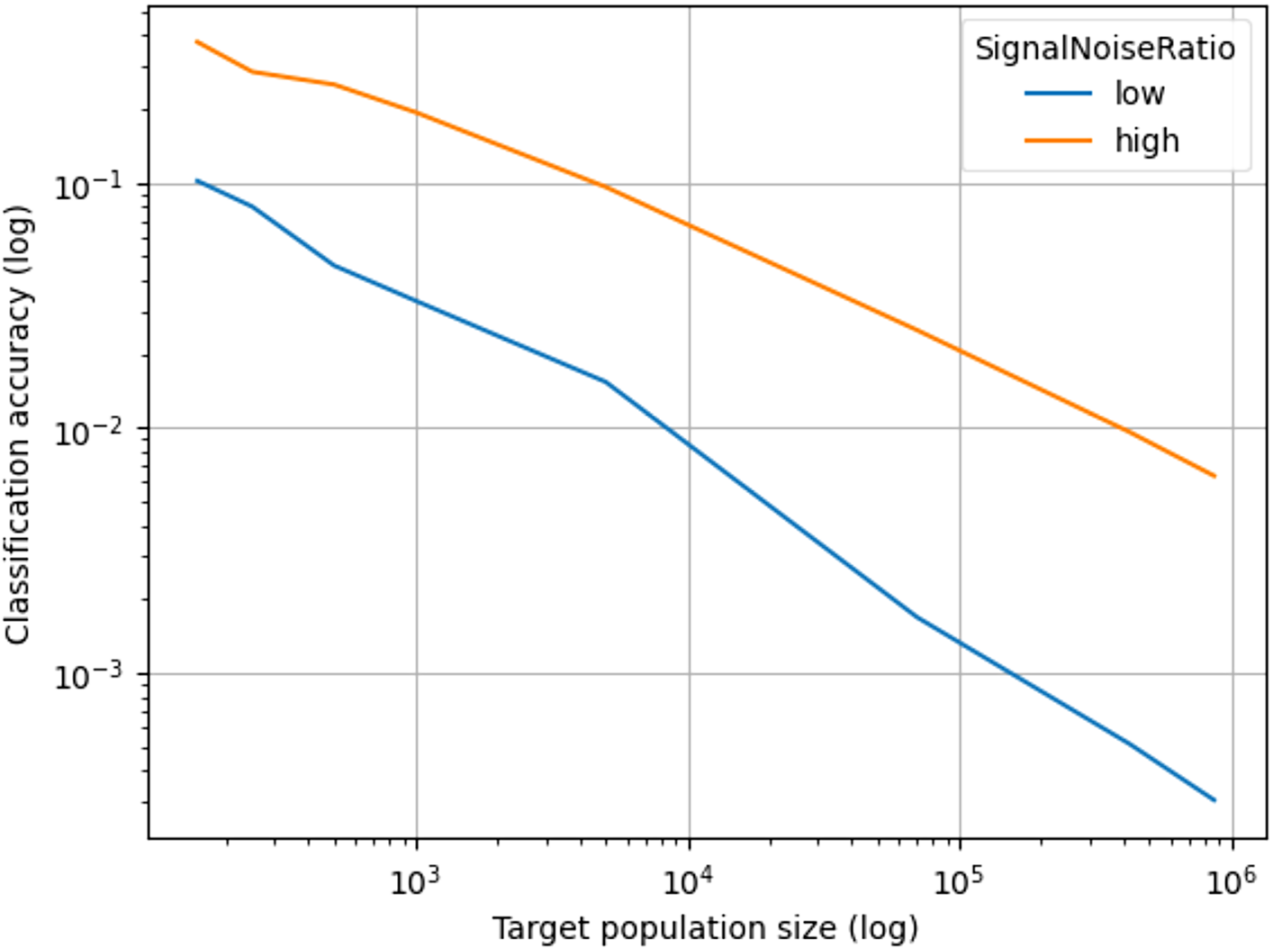
Classification accuracy as a function of the target population, from 157 (the population size used bySchwarz et al. (2021))to 865,000. Results are presented on a log-log scale to allow better visualization of small accuracy values.

Figure 1presents the changes in identification accuracy results from the simulations. For the higher signal-noise simulation (matching subjects’ photos with refaced structural MR images), identification accuracy dropped from 37.6% for the initial population size of 157 to 8.6% at a population size of 6,500; to 2.4% at 70,000; to 0.9% at 423,000; and to 0.6% at 865,000. For the lower signal-noise simulation (matching subjects’ photos with defaced structural MR images), identification accuracy dropped from 9.6% at the initial population size of 157 to 0.8% at a population size of 6,500; to 0.2% at 70,000; to 0.05 % at 423,000; and to 0.03% at 865,000 (Table 1). The relationship between accuracy and population size is roughly linear in log-log space, consistent with theoretical results.

**Table 1.** Reidentification performance across different target population sizes with additional demographic information available.

		Size of population				
		157 ( Schwarz et al., 2021 )	6,500 (narrow age range, gender & race)	70,000 (narrow age range & gender)	423,000 (broad age range & gender)	865,000 (broad age range only)
Reidentification performance	Higher SNR Simulation (Refaced Images)	37.6%	8.6%	2.4%	0.9%	0.6%
	Low SNR Simulation (Defaced Images)	9.6%	0.8%	0.2%	0.05%	0.03%

The results showed that for defaced images, the reidentification accuracies remain substantially low even with additional demographic information. Yet in cases where the attacker is highly motivated and skilled in MR image processing to be able to reface defaced structural images, the accuracies can increase to concerning levels, especially when information on age, gender, and race/ethnicity is all available (8.6%).

## 4 Regulatory implications of reidentification via face recognition in neuroimaging data

Given the results of the simulation analysis, we will examine whether this updated likelihood of reidentification would place defaced neuroimaging data out of compliance under the prominent US regulatory standards on data deidentification. Here, our analysis focuses on US federal regulations—the Common Rule and HIPAA, but there could be other additional state- or institutional-level requirements for data deidentification. For comparison purposes, we will also discuss the implications of reidentification using face recognition under the EU’s privacy regime. Our analysis suggests that defaced neuroimaging data would still meet the regulatory requirements for data deidentification under the current US regulatory regime.

However, it should be acknowledged that a risk analysis is not conducted in a vacuum, and the likelihood of reidentification is just one of the factors that should be considered. For example, when assessing the privacy risk associated with data sharing, we should take into account whether there is other sensitive health or personal information shared with the data. Whether the institutions of data recipients have an adequate organizational data protection system is another critical factor, because data deidentification can offer a sound basis for regulatory compliance only if it is implemented in concert with appropriate administrative, physical, and technical controls addressing privacy and security. Some of these other factors will be discussed in the next section analyzing the new NIH Data Management and Sharing Policy and its supplemental information, which provide best practices for the protection of subject privacy beyond simply satisfying regulatory requirements for data deidentification.

### 4.1 Common rule

One of the main regulatory sources for our analysis is the Common Rule, the federal regulations codified by the US Department of Health and Human Resources (HHS) that were adopted by 20 federal agencies (U.S. Department of Health & Human Services (HHS), 2008). It has been operating as the standard for ethical conduct of federally funded biomedical and behavioral research involving human subjects,^3^laying out two main requirements for the protection of human subjects—informed consent and IRB review.

#### 4.1.1 Regulatory standards/requirements for data deidentification

Under the Common Rule, secondary analysis on shared human subject data that contain identifiable private information is considered human subject research and should adhere to the Rule’s two requirements. Identifiable private information is defined as private information for which the identity of the subject is or may readily be ascertained by the investigator or associated with the information (45 CFR §46.102 (e)(5)). The Common Rule does not define the term “readily ascertainable,” and it is left to individual IRB’s discretion to interpret and apply this standard (Meyer, 2020).

Under certain circumstances, secondary research on identifiable private information could be exempted from the Rule; for example, if the data source is publicly available (45 CFR §46.104 (4)(i)) or the investigator recorded the information without identifiers and does not contact or reidentify subjects (45 CFR §46.104 (4)(ii)). Another exemption for secondary research on identifiable private information is to obtain broad consent, which is a new type of consent adopted in the 2018 revision of the Rule. This exemption permits researchers to obtain broad consent for the storage, maintenance, and secondary research use of identifiable information; secondary research on data that are collected under broad consent do not require additional consent, as long as additional conditions are met, including limited review by an IRB (45 CFR §46.104(8)).

On the other hand, research involving data that are not individually identifiable falls outside the scope of human subject research, and thus, researchers can share and analyze the data without additional consent from the subjects and the IRB review.^4^The Common Rule does not specifically define what is required to render data not individually identifiable. Yet, according to guidance from the HHS Office for Human Subject Research Protection (OHRP), data are not individually identifiable if identifying information (such as name and social security number) has been coded (e.g., replaced with a number, letter, symbol, or combination thereof) and investigator(s) cannot readily ascertain the identity of the subjects to whom the data pertain (Office of Human Research Protections (OHRP), 2008). The identity of the subjects cannot be readily ascertained by the investigator(s) when the release of the key to decipher the coding system is prohibited (1) under an agreement between the investigator(s) and the holder of the key or (2) under IRB-approved written policies, operation procedures for a data repository, or other legal requirements.

#### 4.1.2 Current practice of neuroimaging data sharing under the common rule

For external sharing of neuroimaging data either between individual researchers/institutions or through data repositories, it is a common practice for researchers to code the metadata to redact identifying information and deface structural images to prevent reconstruction of face-recognizing information comparable to that of standard photographs. According to a recent survey study of principal investigators of active NIH grants in neuroscience (Hendricks et al., 2022), most investigators thought deidentification of data protects privacy to a great extent (82%) and almost all reported always deidentifying their individual-level research data before sharing (94%) (Hendricks et al., 2022).^5^Another survey study of neuroimaging researchers found that 65% of the respondents prefer to share their data with other researchers under a data sharing agreement (Paret et al., 2022). The agreements between individual researchers/institutions commonly have a clause that prohibits the recipient from attempting to reidentify data subjects.

Most of the existing neuroimaging data repositories require data to be deidentified before sharing and/or ask the recipients to sign a data use agreement, which includes the prohibition of data reidentification (Jwa & Poldrack, 2022a). Some repositories like the National Institute of Mental Health Data Archive (NDA) explicitly require data to be not individually identifiable following the Common Rule standard. Its Data Sharing Terms and Agreements provide that “[a]ll data made available for public use via NDA will be deidentified data, such that the identities of participants cannot be readily ascertained or otherwise associated with the data by NDA staff or secondary data users (National Institute of Mental Health Data Archive (NDA), 2020).” Others impose more specific requirements to make data not individually identifiable under the Rule. For example, the Washington University-University of Minnesota Consortium of Human Connectome Project clearly states in its open access data use terms that “under no circumstances the code that would link these data to identifiable information be given to the recipient, nor will any additional information about individual human subjects be released to the recipient (WU-Minn HCP Consortium, 2013).” OpenNeuro (openneuro.org), a fully open platform that shares publicly available data, further requires researchers to destroy any key linking the personal identity of research subjects to the codes used in the data set as a condition to upload the data set.

The current common practice of neuroimaging data sharing, including redaction of identifiers through a coding system (defacing) and use of terms and agreements to prevent reidentification, would make the shared data qualify as not individually identifiable under the OHRP guidance. Therefore, in principle, secondary analysis on the shared data would fall outside the Rule, unless there are any additional requirements from individual IRBs.

#### 4.1.3 Implications of face recognition

The OHRP guidance on identifiability of data only focuses narrowly on whether the data can be reidentified through coding systems, without regard to other emerging reidentification techniques (Meyer, 2020;Office of Human Research Protections (OHRP), 2008). However, advanced face recognition techniques raise the possibility that the coded neuroimaging data could be directly linked back to individual subjects without needing to decipher a linking code. Even if identifiers in the metadata are removed and replaced with the code, individual subject’s identities can potentially be established via face recognition by matching the facial features reconstructed from the data with publicly available photos of subjects. In other words, destroying the key to the code or prohibiting the release of the key may not guarantee that the data are sufficiently protected from future reidentification. Taking into account the novel technological threat to privacy, the 2018 revised Common Rule requires federal agencies adopting the Rule to reexamine the meaning of identifiable private information at least every 4 years upon consultation with appropriate experts, including experts in data matching and reidentification (§46.102 (e)(7)).

Nevertheless, at least under the Common Rule’s current standard, defaced neuroimaging data with coded identifiers would still be considered not individually identifiable. Because the OHRP guidance was not formulated anticipating the potential for reidentification via face recognition, researchers and IRBs would need to go back to the provisions in the Common Rule to determine the identifiability of the defaced neuroimaging data. As discussed above, identifiability of private information is vaguely defined in the Rule (§46.102(e)(5)), and in part due to this—probably intentional—uncertainties, “the Rule’s bar for rendering data nonidentifiable is fairly low (Meyer, 2020).” The core question here is whether the identity of the subject is readily ascertainable. Again, the Rule does not define the term “readily ascertainable,” but the results of our simulation analysis demonstrate that despite the growing privacy concerns around face recognition technique, the likelihood of reidentification in defaced images is substantially low in the population size large enough to be realistic, even with additional demographic information. Thus, given the Common Rule’s relatively lenient standard, it would be difficult to argue that this low real-world likelihood of reidentification would make the identities of subjects “readily ascertainable.”

### 4.2 Health insurance portability and accountability act of 1996 (HIPAA)

Another relevant regulatory source for this analysis is the Health Insurance Portability and Accountability Act of 1996 (HIPAA) (U.S. Department of Health and Human Services (HHS), 2003). The HIPAA Privacy Rule is a federal regulation that establishes national standards to protect individuals’ medical records and other health information.

#### 4.2.1 Regulatory standards/requirements for data deidentification

The Privacy Rule standards address the use and disclosure of individually identifiable health information held or transmitted by a covered entity.^6^This information is called protected health information (PHI). Individually identifiable health information is a certain type of health information,^7^including demographic data, that identifies the individual or for which there is a reasonable basis to believe that it could be used to identify the individual (45 CFR §160.103).

Unlike the Common Rule, which focuses on the protection of human research subjects, the HIPAA’s central purpose is to protect privacy and confidentiality of health data. In this context, privacy means a right to control when, how, and to what extent one’s health information can be shared with others, and confidentiality refers to the right to prevent further disclosure of health information one has chosen to divulge to another (e.g., physicians or researchers). The Privacy Rule standards are built upon, and not intended to supersede, the Common Rule’s human subject protection; a researcher who collects PHI from human subjects as part of a covered entity must comply with both the Common Rule and the HIPAA Privacy rule. However, the HIPAA guidelines are also commonly used even outside of HIPAA-covered entities, in part due to widespread confusion among researchers regarding the scope and coverage of HIPAA.

The basic principle under the Rule is that a covered entity may not use or disclose PHI, except either when the Rule permits/requires or when there is a written authorization from the individual who is the subject of the PHI (U.S. Department of Health and Human Services (HHS), 2003). For example, the Rule permits the use or disclosure of PHI for research purposes, without an individual’s authorization, if the covered entity obtains an alteration or waiver of individual authorization for the use or disclosure of PHI for research purposes approved by an HIPAA Privacy Board or IRB (45 CFR §164.512(i)(i)) or if the use or disclosure of PHI is solely for reviews preparatory to research or for research on PHI of decedents (45 CFR §164.512(i)(ii), (iii)).

However, the Rule also allows a covered entity to use PHI to create information that is not individually identifiable, which is referred to as deidentified health information. Deidentified health information neither identifies nor provides a reasonable basis by which to identify an individual, and there are no restrictions on the use or disclosure of deidentified information by a covered entity (45 CFR § 164.502(d)(2)). The process of deidentification is intended to mitigate privacy risks to individuals and thereby supports the secondary use of data (e.g., for research studies and policy assessment) (Office for Civil Rights (OCR), 2012).

Whereas the Common Rule provides only a general definition of private information that are not individually identifiable, the Privacy Rule sets out two specific standards to deidentify PHI. One is formal determination by a qualified expert that there exists a very small risk that the information could be used to identify an individual to whom the information pertains based on generally accepted statistical and scientific principles and methods (Expert Determination; 45 CFR §164.514(b)(1)). The other is the removal of 18 unique identifiers^8^from PHI, given that a covered entity does not have actual knowledge that the information could be used alone or in combination with other information to identify the subject of the information (Safe Harbor; 45 CFR §164.514(b)(2)). Due to their clarity and simplicity, these standards, particularly the Safe Harbor provision, have been commonly used by researchers regardless of whether they are conducting research as a part of an HIPAA-covered entity.

#### 4.2.2 Current practice of neuroimaging data sharing under the HIPAA privacy rule

In determining which identifiable information should be removed (or coded) prior to sharing, neuroimaging researchers have commonly followed the Safe Harbor standard. All unique identifiers are stripped from the data, including facial features in structural scans, which can be counted as one of the 18 HIPAA identifiers—full face photographic images and any*comparable*images. Some neuroimaging repositories, such as International Neuroimaging Data-Sharing Initiative (International Neuroimaging Data-Sharing Initiative, n.d.) and OpenNeuro (openneuro.org), specifically require the shared data to be deidentified in accordance with the HIPAA Privacy Rule’s standards. Yet in cases wherein simply redacting the identifiers would still not be sufficient to prevent the reidentification of data (e.g., data collected from family studies or studies on geographically limited populations), researchers have implemented additional safeguards, such as controlling access to data or applying statistical methods to further reduce the risk (Brakewood & Poldrack, 2013).

#### 4.2.3 Implications of face recognition

It is important to note that Safe Harbor requires not only the removal of unique identifiers but also the absence of actual knowledge by the covered entity that the remaining information could be used to identify individuals. Particularly relevant to our analysis, this raises the question of whether being aware of recent studies on face recognition could constitute having actual knowledge that defaced neuroimaging data could still be used to reidentify data subjects (45 CFR §164.514(b)(2)).

The guidance from the Office for Civil Rights (OCR) within HHS, which has responsibility for implementing and enforcing the HIPAA Privacy Rule, sheds light on this question (Office for Civil Rights (OCR), 2012). It states that mere knowledge of studies about methods to reidentify health information does not necessarily count as actual knowledge under the provision. It further explains that covered entities are not expected to presume that all potential recipients of deidentified data have such capabilities to reidentify data using these methods, which is not consistent with the Safe Harbor standard’s intent to provide a simple way to deidentify health information.

In other words, even if researchers know of the specific studies about face recognition to reidentify neuroimaging data, this will not affect achieving data deidentification under this standard. It may be possible that an increase in the awareness among researchers and policymakers concerning the likelihood of reidentification posed by face recognition could lead to a different interpretation of this second requirement. Yet, considering the low likelihood of real-world reidentification of defaced images via face recognition as shown in our simulation analysis, it is unlikely this would happen in the near future for most studies of larger populations.

The other HIPAA deidentification standard—expert determination—is rarely used for neuroimaging data, but it might be useful to examine the potential implication of face recognition in complying with this standard. The OCR guidance states that there is no explicit numerical level of the likelihood of reidentification deemed to universally meet the “very small” level indicated in the standard (Office for Civil Rights (OCR), 2012). The rationale behind using this rather vague term is that the ability to identify subjects of PHI can vary across anticipated recipients of the information depending on multiple factors. When evaluating the probability of reidentification, an expert often considers three factors: (i) whether the information features in deidentified data are individually unique or distinguishing, (ii) whether external data sources contain the individuals’ identifiers and their unique features, and (iii) whether there is a mechanism to relate the deidentified data to these external data sources.

The remaining facial features in defaced neuroimaging data could be individually unique and distinguishing. Publicly available facial photos from the internet or social network service could also count as external data sources that can be linked to defaced data to reveal the identity of data subjects. However, as the real-world matching accuracy for defaced images is very low, it would be difficult to infer that face recognition algorithm provides a reliable mechanism by which defaced data can be linked to publicly available photos. Therefore, the low likelihood of reidentification posed by face recognition would most likely not render defaced neuroimaging data noncompliant under the expert determination standard at present.

### 4.3 European Union’s general data protection regulation (GDPR)

The General Data Protection Regulation (GDPR) in the EU is the most stringent privacy regime in the world (European Parliament & Council of the European Union, 2016). Unlike the United States where there is a patchwork of sector-specific privacy laws (e.g., HIPAA for health data) without an umbrella federal privacy regulation, GDPR regulates the collection, processing, sharing, and storing of personal data, which is broadly defined as any “information relating to an identified or identifiable natural person (‘data subjects’) (Art. 4(1)).” It prevents the processing of personal data unless there is a lawful basis, for example, when the data subject has given consent to the processing (Art. 6(1)). GDPR also stipulates a number of individual rights of data subjects, notably including the right to erasure (“right to be forgotten”) (Art. 17), with certain exemptions when processing data for research-related purposes (Art. 9 (2)(i),(j)).^9^

The protection under the GDPR does not apply to anonymized personal data, but the GDPR standard for anonymization is far more stringent than the HIPAA’s deidentification standard. Personal data that have undergone pseudonymization, which means the processing of personal data to make the data cannot be attributed to a specific data subject without the use of additional information (e.g., redacting direct identifiers and replacing them with a code), still falls under the scope of the GDPR (Vokinger et al., 2020). On the other hand, anonymization is “an irreversible process” that renders the subjects of personal data not to be identifiable or no longer be identifiable (D. Eke et al., 2021; GDPR recital 26).

According to Recital 26, in determining whether data are identifiable, “account should be taken of all the means reasonably likely to be used, such as singling out.” Several factors, such as costs of and the amount of time required for identification and the available technology, should be considered to evaluate the reasonableness of the means to be used for reidentification. Identifiability of data must be decided on a case-by-case basis, and this potential room for interpretation “leads to serious uncertainties in practice (Vokinger et al., 2020).” In fact, under the GDPR’s stringent standards, “nowadays nearly all processes performed on raw and derived data that are associated with a specific natural person can at most only be classified as “pseudonymized” (D. Eke et al., 2021).”

Deidentifying neuroimaging data through the above-stated methods—removal of identifiers in the metadata through a coding system and defacing of structural scans—would be considered pseudonymization of data at best under the GDPR’s definitions. In other words, neuroimaging data currently shared through data repositories will be largely treated as fully identifiable personal data and secondary research on the data must comply with all GDPR requirements and limitations.^10^Thus, the novel techniques for reidentification, such as face recognition, which would only increase—although minimally—the identifiability of defaced neuroimaging data, would not affect the status of the data as pseudonymized data under the GDPR.

## 5 The new NIH data management and sharing (DMS) policy

Regulatory requirements only specify minimally required privacy protection, and thus, complying with these requirements does not necessarily mean that neuroimaging researchers meet their broader ethical duty to protect research subjects. As privacy becomes a global imperative in the surge of big data analytics, there has been a growing awareness of the need for more rigorous data protection in the context of data sharing.

Recognizing this need, NIH recently released supplemental information to its new data management and sharing (DMS) policy to guide researchers in addressing privacy considerations when sharing human research participant data. The new DMS policy, which became effective in January 2023, is intended to reinforce the NIH’s long-standing commitment to data sharing (National Institutes of Health (NIH), 2020a). It requires submission of and compliance with data management and sharing plans for research funded or conducted by NIH that results in the generation of scientific data.

The supplemental information to this new policy outlines best practices and points to consider for the responsible sharing of scientific data consistent with protecting research participant privacy (National Institutes of Health (NIH), 2022). This information is not creating new binding rules, but it is expected to operate as an authoritative source of guidance not only for the awardees of NIH grants but also for any researchers in the United States who want to share human participant data. Yet because it only provides general recommendations without regard to specific types of data, it would be helpful to contemplate its implications for the sharing of neuroimaging research data.

The supplemental information first recommends deidentification of research data to the greatest extent that it maintains sufficient scientific utility. Researchers should rely on the standards for identifiability in both the Common Rule and the HIPAA, regardless of whether these rules apply to the sharing, disclosure, or subsequent use of their data. NIH further underlines that even when data are deidentified under the Common Rule and HIPAA standards, there may still be a remaining likelihood of re-establishing the identities of subjects, and researchers should consider the remaining risks and implement strategies to avoid reidentification (e.g., modify the data or sharing data only through controlled access).

As discussed in the previous section, neuroimaging researchers have commonly referred to both the Common Rule and the HIPAA in determining the identifiability of their data (even when they are not a part of a HIPAA covered entity). Again, the conventional deidentification methods used for open sharing of neuroimaging data, such as redaction of direct identifiers and removal of facial features in the data, meet the current standards of identifiability in these regulations, even considering the likelihood of reidentification via novel tools such as face recognition. Researchers should keenly follow new development of reidentification technology to re-evaluate the privacy risk and develop countermeasures to reduce the risk. Yet given the low likelihood of reidentification demonstrated in this study, opting out to fully controlled access for neuroimaging data would substantially limit the benefits of open data sharing without resulting in meaningful enhancement of privacy protection.

Second, it is recommended to use data sharing/use agreements, preferably standardized, when sharing data through data repositories even if the data are deidentified. Agreements for submitting data to repositories should include assurance that an institutional body has evaluated the risks of data sharing, that sharing is consistent with informed consent, and that the privacy measures in place are appropriate. Data use agreements for the data recipients should delineate the responsibilities and restrictions in the use of shared data (e.g., limitations on sharing and future use of the data, responsibilities regarding privacy and confidentiality, prohibition of participants’ reidentification, and methods used to deidentify data and any relevant risk assessments).

Existing neuroimaging data repositories have implemented standardized data sharing/use agreements for researchers to deposit or gain access to data (Jwa & Poldrack, 2022a). Repositories that have data sharing agreements (or policies) (DSAs) require researchers to deidentify data before sharing and often specify the standards and methods to be used for deidentification. Some DSAs explicitly state that data should be shared in accordance with informed consent and/or with the approval of the Institutional Review Board (IRB) (National Institute of Mental Health Data Archive (NDA), 2023a), but it is not clear this term includes that the risks of data sharing or adequacy of privacy measures should be evaluated by an institutional body. Almost all data use agreements (DUAs) for neuroimaging data repositories do prohibit attempts to reidentify or recontact data subjects and require all users having access to shared data to comply with the terms of DUA (Jwa & Poldrack, 2022a). Some repositories restrict redistribution of shared data (National Institute of Mental Health Data Archive (NDA), 2023b;Alzheimer’s Disease Neuroimaging Initiative (ADNI), 2024), but these standardized DUAs generally do not include specific sharing and use limitations for individual data sets. Detailed deidentification methods and assessment of privacy risks for each data set to which access is requested also do not appear in the DUAs.

Finally, the supplemental information encourages researchers to understand the applicability of a variety of relevant laws, regulations, and policies at the federal, tribal, state, and local levels that impose obligation on the disclosure and use of scientific research data. Per this recommendation, neuroimaging researchers who want to share their data should not only comply with the HIPAA and Common Rule, which only serve as a floor of privacy protections, but also consider the regulatory requirements and restrictions at a different level, which often materially diverge from those under the federal laws and regulations and are permitted to add further requirements.

Along with these best practices, the supplemental information moreover provides several factors to account for determining whether to designate the data for restricted access, as the new DMS policy strongly suggests to consider controlling access to human participant data, even if the data are deidentified and lacking explicit limitations on subsequent use.

First, researchers should consider whether there are explicit limitations on subsequent use of data imposed by laws, regulations, policies, informed consent, or other agreements. The second factor is whether data could be considered sensitive, such as the cases wherein the data contain potentially stigmatizing traits or illegal behaviors that could cause group harm or be used for discriminatory purposes or when the data include unique traits of participants that increase the likelihood of reidentification. Third, researchers may not openly share their data if the data cannot be adequately deidentified to meet existing regulatory standards or if the possibility of reidentification cannot be sufficiently reduced even after the data are deidentified. Finally, access to data may need to be restricted if new emerging approaches or technologies could pose increased privacy risks to participants.

It is apparent that neuroimaging data with explicit use limitations should be shared through controlled access, for example, when the informed consent clearly states that a participant’s data will not be shared with researchers or institutions. For neuroimaging data that contain highly sensitive information as described in the supplemental information, researchers may want to share their data through controlled access. However, as acknowledged in the information (National Institutes of Health (NIH), 2022), even if data are sensitive, it may be possible to deidentify the data in ways that would allow appropriate sharing. Thus, researchers should critically evaluate the sensitive nature of their data and associated privacy risk, including the likelihood of reidentification, before choosing to restrict the access to the data. In addition, it has been argued that neuroimaging data would no longer be able to be sufficiently deidentified for open sharing due to the novel techniques like face recognition and the practice of making the data publicly available may well be illegal and would not be tenable any longer (D. Eke et al., 2021). Despite this profound concern, our technical and regulatory analyses showed that the likelihood of reidentification in defaced images via face recognition in a real-world setting is still very low and would not put deidentified neuroimaging data out of regulatory compliance at least in the United States. As an extension of these results, it would also be difficult to argue that face recognition should be counted as an emerging reidentification technique, one that will increase the privacy risk to an extent that justifies controlled access to deidentified neuroimaging data.

Alternatively, according to the supplemental information, researchers may share scientific data without access controls when there is explicit consent from participants to share their data openly or when scientific data are deidentified and an institutional review has determined that the likelihood of reidentification is very low. Data sharing should be planned prospectively from the inception of a research study, including the process of developing an informed consent form.

Open platforms (e.g., OpenNeuro) have recommended researchers to use a consent form intended for public sharing of deidentified data, such as Open Brain Consent, which provides model forms that meet different regulatory standards in the United States and EU (Bannier et al., 2021). In terms of the institutional review of the privacy risk associated with data sharing, the supplemental information does not specify an institutional office or component tasked with this review. The IRB could be a good candidate, but it may not have the needed expertise to evaluate the risk, not to mention the concern about burdening it with an additional task on top of an already substantial workload.

## 6 Discussion

The advancement in computational tools and machine-learning algorithms has greatly facilitated the analysis of shared neuroimaging data, but at the same time, concerns have been raised that they could be used to undermine data privacy by reestablishing the subjects’ identities from the data that are thought to have been deidentified. In this study, we first examined the likelihood of reidentification in defaced neuroimaging data using face recognition to test the generalizability of the matching accuracies reported inSchwarz et al.’s (2021)study. The simulation analysis showed that the likelihood of reidentification in defaced images decreases substantially as the pool of potential matches increases to the realistic size of the population, even with additional demographic information (age, gender, or race/ethnicity) (0.03%–0.8%). Although less likely, if we assume a highly motivated and skilled attacker capable of imputing redacted facial features in defaced images with a population-average face template, the reidentification accuracies could reach worrisome levels (0.6%–8.6%) depending on demographic information available. These results suggest that given the efficacy of defacing in preventing subject reidentification, in spite of the new threat posed by advanced face recognition, defacing should be considered best practice for data meant to be shared openly.

Our threat model assumes a sample-to-population problem (reidentification of a subject using neuroimaging data by matching reconstructed facial features from the data with publicly available facial images of a potential target population), but one can also imagine a scenario whereby an attacker already has a known or suspected individual to be a subject of a dataset and then attempts to reidentify this target individual by matching the target’s photos with reconstructed facial features from the datasets (Clunie et al., 2023). In this case, the size of the pool of the potential target is the size of the dataset at hand, which would be far smaller than that of the example we used to test our threat model (6,500, 70,000, 423,000, and 865,000). For example, ADNI has approximately 5,000 participants across roughly 50 centers (Alzheimer’s Disease Neuroimaging Initiative (ADNI), n.d.). If the target could be localized to a major geographic region (i.e., narrowed down to a single site), the values would be closer to 100 (5,000/50), which is much closer to the sample size inSchwarz et al.’s (2021)study (N = 157).

Aside from the technical feasibility of reidentification, it would also be important to consider the potential attackers’ incentives as well as the costs to reidentify data (Meyer, 2018). For example, recent literature on genetic data privacy has suggested that the information about an individual that can be learned from reidentified genetic data (e.g., genetic traits and predispositions) might be more easily available through other sources (Clayton et al., 2019), and the cost of attempting to reidentify the data may make the attack not worth pursuing (Wan et al., 2015,^2017^). Similarly, the personal information that can be extracted from neuroimaging data and associated metadata in cognitive neuroscience or psychological science research could largely be obtained from analyzing other personal or biometric data (e.g., internet search history, GPS tracking, or data from social media platforms) without going through the technical challenges in handling neuroimaging data. In fact, there has been no actual report of research data reidentification, except the circumstances wherein the data are used by privacy researchers to test the practicability of reidentification (Clunie et al., 2023;Meyer, 2018).

It should be acknowledged that there are baseline probabilities of reidentification through more generic ways in any personal or medical data that reach the public sphere. It has been reported that a handful of simple demographic variables is often sufficient to uniquely identify an individual (Sweeney, 2000). Using a generative graphical model,Rocher and colleagues (2019)also showed that individuals can be accurately reidentified even from heavily incomplete deidentified socio-demographic, survey, and health datasets. However, although simple demographic data have been shared in association with medical research data for several decades, there are few known incidents of threat actors exploiting these data—and again, no known successful reidentification attack in neuroimaging data—outside of academic demonstrations.

Following the simulation analysis, we further examined whether this novel threat of reidentification would put defaced neuroimaging data out of compliance with the deidentification standards under the US regulatory regime. The analysis of the relevant laws and regulations, such as the Common Rule and HIPAA, suggests that conventional deidentification methods used for open sharing of neuroimaging data, including defacing, would still provide sufficient protection for privacy as required under the current regulatory standards. Given the low likelihood of real-world reidentification, applying a face recognition algorithm would hardly make the identity of the subjects of defaced neuroimaging data readily ascertainable under the Common Rule. It is also unlikely that the novel threat posed by advanced face recognition would affect achieving deidentification of neuroimaging data under HIPAA’s two standards—expert determination and safe harbor methods.

In addition, the current practices of neuroimaging data sharing, including the deidentification of data, use of data sharing/use agreements in the repositories, and compliance with regulatory requirements for privacy protection, are overall well-aligned with the best practices the new NIH DMS policy recommends. The policy and supplemental information encourage researchers to consider controlling access to shared human participant data even when not required. When there are explicit legal or regulatory restrictions or the data contain highly sensitive information about the subjects, sharing and use of the data should be limited. However, except in these circumstances, neuroimaging data can still be shared openly in compliance with the NIH’s guidance by obtaining an appropriate informed consent from the subjects or a determination of low risk by an institutional body.

### 6.1 Future directions of open data sharing in neuroimaging

Open data sharing allows the data to be accessible to the largest possible number of researchers and citizen-scientists (Markiewicz et al., 2021), compared with restrictive sharing, such as limiting access only to certain groups of individuals (e.g., qualified researchers) or controlling subsequent use of data through DUA and/or other mechanisms (e.g., review of planned secondary research by a data access committee) (Alzheimer’s Disease Neuroimaging Initiative (ADNI), 2024). Openly sharing data thus maximizes transparency, public accountability, and the potential of data to generate new scientific discoveries.

The open science practice is predicated on the assumption that deidentification of data ensures adequate protection for subjects’ privacy and the confidentiality of the data. The emerging tools and algorithms and recent demonstrations of data reidentification via these tools and algorithms seem to threaten this assumption. Various policy and organizational measures have been proposed to control access to and use of neuroimaging data (D. Eke et al., 2021;D. O. Eke et al., 2022;Goering et al., 2021;Ienca et al., 2022), but outright restrictions could significantly hinder open science practice in the field. More importantly, open sharing and restrictive sharing should be viewed on a spectrum, rather than as mutually exclusive options. The level of privacy risk differs across neuroimaging datasets, as the nature and sensitivity of the information in the datasets vary. Knee-jerk restrictions would thus unduly limit the benefits of open sharing. Policy and regulations should aim to make research data publicly available to the extent possible, and any limitations on sharing and secondary use of data should be carefully calibrated, corresponding to the specific risk associated with individual data sets to avoid chilling effects on open sharing (Clunie et al., 2023).

Moreover, in determining the overall privacy risk of neuroimaging data, we should consider not only the likelihood of reidentification but also the magnitude of harm that would be incurred to subjects should reidentification occurs (Meyer, 2018). As our simulation analysis has demonstrated, the likelihood of reidentification of defaced neuroimaging data is still very low even against the attack using a cutting-edge face recognition technique (0.03%–0.8%); but for refaced data, the matching accuracy increased up to 8.6% when additional demographic information is available. Yet these likelihoods do not necessarily equate to the privacy risks, and the potential harms to subjects resulting from the reidentification should also be included in the calculation of the risks.

Here, the harms associated with neuroimaging data are expected to be mainly informational and discriminatory. Most neuroimaging studies in cognitive/psychological neuroscience do not contain metadata regarding the subjects’ medical conditions, which could, if misused, put the subjects in peril (e.g., in a health insurance or employment context). The major aim of these studies is rather to investigate neurobiological mechanisms underlying cognitive and psychological processes using various experimental tasks. Mere disclosure of neuroimaging data from these studies, along with other associated information (e.g., cognitive task performances), would be unlikely to cause material or reputational harm to the subjects. Therefore, it can be said that the overall privacy risks associated with these data—the projected minimal harm discounted by the likelihood of reidentification—is extremely low at least for the near future.

However, neuroimaging data from clinical neuroscience research do contain highly sensitive personal or medical information (e.g., a biomarker of a neurodegenerative disorder or diagnosis of a rare disease). These data would deserve more heightened protection because the potential harm occurring from the malicious use of the data could be substantial, even if the likelihood of reidentification is still low. Yet imposing rigorous restrictions on access to and use of the data will not be able to completely prevent a reidentification attack and the resulting unwanted disclosure of data. Moreover, the harm to the subjects will only materialize when the attacker actually exploits the sensitive information in ways detrimental to the subjects. We proposed elsewhere that a legal prohibition of the misuse of neuroscience data—including neuroimaging data—similar to the Genetic Information Non-Discrimination Act (GINA) in the United States would provide a more ultimate protection against the potential harm to the subjects beyond controlling the sharing and use of the data (Jwa & Poldrack, 2022b).

In addition, evaluating the privacy risk associated with a particular neuroimaging data set can be a daunting task because it requires a sound understanding of both the technical and regulatory aspects of the risk. It has been largely left under the discretion of the researchers who want to share their data, with some guidance from their institutions (e.g., the IRB or privacy office). As the reidentification techniques as well as the regulatory landscape have been evolving rapidly, it becomes more difficult to accurately assess the risk and ensure compliance with legal and regulatory requirements, including the funding agencies’ data sharing requirements.

Researchers call for additional guidance or best practices for sharing human neuroscience data, for example, regarding standards of data deidentification, who can have access to data, and what data should or should not be shared (Hendricks et al., 2022). The recommendations outlined in the supplemental information to the new NIH DMS policy could provide some preliminary guidance (National Institutes of Health (NIH), 2022), but more detailed best practices particularly focused on neuroimaging data would be needed to better inform researchers of the standards and due diligence for sharing their data. Academic societies and professional associations in related fields could play a leading role in developing the best practices for neuroimaging data.

The institutional review of privacy risk recommended under the NIH’s supplemental information could also be beneficial to promoting the responsible sharing of data (National Institutes of Health (NIH), 2022). According to the supplemental information, no specific institutional office or component is suggested to conduct these reviews, as long as the individual(s) involved possess the appropriate expertise and institutional role(s). However, regardless of which institutional body takes responsibility, it would be critical to have scientifically and technically informed reasonable standards in examining the risk to avoid inconsistent or overly risk-averse determination.

On the side of data repositories, there is a range of options available regarding the types of sharing (e.g., fully open sharing, tiered approach depending on the risk, and controlled access through the DAC approval) and other restrictions (e.g., prohibition of identification or limitations on future use). This allows researchers to choose an appropriate repository for their neuroimaging data given the risk level (Jwa & Poldrack, 2022a). Another supplemental information to the NIH’s DMS policy that sets forth generally desirable characteristics of data repositories could provide useful guidance for neuroimaging researchers (National Institutes of Health (NIH), 2020b).^11^The repositories should also put an effort to implement these characteristics. In addition, as a significant portion of data sharing is still occurring through a direct personal request between individual researchers or research groups (Paret et al., 2022), best practices for neuroimaging data sharing should include considerations relevant to these circumstances, such as how to devise and negotiate a data use agreement pursuant to direct requests.

Finally, keeping up the advances in AI and machine-learning tools and algorithms for data reidentification, we expect that more sophisticated technical solutions to counter these novel threats would also be developed. Federated computing tools to analyze large-scale neuroimaging data in multi-site research without actually pooling raw-individual data (Plis et al., 2016) or cutting-edge privacy preserving methods, such as differential privacy, are some of the examples used for sharing and analyzing neuroimaging data. In particular for the technological threat of face recognition techniques, new techniques using adversarial machine learning may be able to fool the face recognition algorithms and substantially reduce the likelihood of reidentification.

## 7 Conclusion

Data sharing has been largely promoted in neuroimaging for the last decade, and as neuroimaging data become more available for secondary analysis, the transparency and reproducibility of neuroimaging research have been greatly enhanced. However, sharing individual-level human subject data requires rigorous privacy measures to protect the subjects. Emerging tools and algorithms have begun to raise doubts about the methods of deidentification used to openly share neuroimaging data that have been considered adequate privacy protection and to question open science practice in the field.

In this study, we examined the likelihood of reidentification via face recognition, one of the most imminent threats to neuroimaging data privacy the literature has raised. Our simulation analysis demonstrated that the likelihood of reidentification in defaced images significantly drops as the potential pool of the population for comparison increases large enough to be realistic, even when additional demographic information is available. This result also suggests that defacing should be considered a best practice for publicly shared datasets. Our regulatory analysis further showed that defaced neuroimaging data would still comply with the standards of identifiability/deidentification required under the US regulatory regime, such as the Common Rule and HIPAA, even considering the novel reidentification tools and algorithms. These results are not meant to discount the need for improved deidentification techniques and other policy and regulatory measures to further protect subject privacy. However, they do suggest the need for a more balanced view of the real-world likelihood of reidentification in neuroimaging data when weighed against the benefits of data sharing and open science practice.

In fact, current practices of neuroimaging data also largely conform to the recommendations for responsible sharing of human subject data outlined in the new NIH DMS policy and supplemental information, which endorse more heightened privacy protections than the regulatory requirements do. Considering the low likelihood of reidentification given the current state of technology, the accessibility of neuroimaging research data to the public should continue to be maximized as much as possible. Implementing restrictions on data sharing and future use of data should be based on sound technical and scientific evidence of risk. Best practices for neuroimaging data sharing on some critical issues, such as evaluation of the likelihood of reidentification, data deidentification, selection of appropriate repositories, and reasonable privacy measures per different levels of sensitivity of the data, would be needed to better inform researchers of the standards and due diligence for sharing their data. In addition, institutional support for researchers to understand the privacy risk associated with data and to devise appropriate protections against the risk (e.g., data use agreement) is called for. Data repositories should also equip themselves with desirable measures and mechanisms for the preservation and sharing of human neuroimaging data. Future development in technical countermeasures to the novel privacy attack would further aid the open sharing of neuroimaging data.

## Data Availability

The script for the simulation analysis in this article is available here:https://doi.org/10.5281/zenodo.10359718; the results can also be viewed inhttps://github.com/poldrack/reidentification-simulations

## Footnotes

1 Schwarz et al. (2021)used FLAIR images to compute the matching accuracies due to these images’ greater effectiveness for facial reconstruction. Subsequently, in their most recent study,Schwarz et al. (2023)tested the recognition rates in other types of MR images, which are more common in publicly shared datasets, such as T1-weighted and T2-weighted images, and showed that if non-defaced, the rates were all similar at 96% to 98%.

2 The population-average face template was created using scans of 177 Mayo Clinic participants ages 30–89 (stratified by age-decade and sex; 120 cognitively unimpaired and 57 with clinically-diagnosed Alzheimer’s disease), imaged using 3D T1-weighted MPRAGE sequences in Siemens Prisma scanners. For a more detailed method used to generate the template and refacing brain images with the template, seeSchwarz et al. (2021).

3 The Common Rule applies to all human subject research conducted, supported, or otherwise subject to regulation by the federal government or otherwise covered by an Office of Human Research Protections (OHRP) approved federal-wide assurance (45 C.F.R. §46.101(a)).

4 However, institutions can implement policies that empower their IRBs to review data sharing and impose more rigorous standards even if research on shared data is not covered by the Common Rule. Thus, researchers should consult with their IRBs when sharing nonidentifiable data.

5 The PIs participated in this survey were investigators in neuroscience, not limited to neuroimaging, and here the term deidentification was defined rather broadly as “removing information that can be used to distinguish or trace an individual’s identity, either alone or when combined with other information.” Thus, it is not clear whether this definition includes defacing of structural MR images.

6 Covered entities under the HIPAA Privacy Rule include health plans, health-care clearinghouses, and those health-care providers that conduct certain health-care transactions electronically (45 CFR §160.103). Research institutions, such as a college or university, could also qualify as covered entities, if they have health-care components that perform covered functions (e.g., providing health care at their medical facilities).

7 Information relates to (a) the individual’s past, present, and future physical or mental health or conditions, (b) the provision of health care to the individual, and (c) the past, present, or future payment for the provision of health care to the individual*and*(i) that identifies the individual or (ii) with respect to which there is a reasonable basis to believe the information can be used to identify the individual. (45 CFR §160.103).

8 The 18 unique identifiers under the Rule include name; all geographic subdivisions smaller than state, including street address, city county, and zip code; all elements (except years) of dates related to an individual (including birth date, admission date, discharge date, date of death, and exact age if over 89); telephone numbers; fax numbers; electronic mail addresses; Social Security numbers; medical record numbers; health plan beneficiary numbers; account numbers; certificate/license numbers; vehicle identifiers and serial numbers, including license plate numbers; device identifiers and serial numbers; web URL; internet protocol (IP) address; biometric identifiers, including finger and voice prints; full face photographic image and any comparable images; and any other unique identifying number, characteristic, or code.

9 GDPR provides special exemptions that allow processing of special categories of personal data, including health data, for scientific research (Art.9(2)(i), (j)). Processing of personal data under these exemptions are subject to appropriate safeguards—technical and organizational measures, including pseudonymization, in accordance with GDPR, for the rights and freedoms of the data subject (Art.89(1)). Moreover, GDPR permits processing of personal data when such processing is not incompatible with the initial purposes of processing, noting that “further processing for archiving purposes in the public interest, scientific or historical research purposes or statistical purposes shall, in accordance with Article 89(1), not be considered to be incompatible with the initial purposes (Art.(1)(b)).” These exemptions also allow EU member states to grant derogations of some of data subject’s rights (Art. 89(2)) for research. However, there has been no effective guidance issued on these provisions to date and no clear permission in the member states’ laws regarding secondary research use of personal data, which has created substantial uncertainty and confusion in the research community (Peloquin et al., 2020). Along with these uncertainties around legal guidance, the harsh penalties for the violation of GDPR, including the liability to individual data subjects (Art. 83), the administrative fines (Art. 83), and other penalties that can be additionally imposed by member states (Art. 84), have further resulted in significant fear of data sharing among researchers and research institutions.

10 Anonymization of neuroimaging data would require more drastic measures beyond the conventional deidentification methods, such as “cross-subject aggregation and randomization procedures,” yet these measures may also reduce the quality or scientific value of the data (Eke et al., 2021).

11 The desirable characteristics for data repositories outlined in the supplemental information are (1) unique persistent identifiers, (2) long-term sustainability, (3) metadata, (4) curation and quality assurance, (5) free and easy access, (6) broad and measured reuse, (7) clear use guidance, (8) security and integrity, (9) confidentiality, (10) common format, (11) provenance, and (12) retention policy. Additional considerations for repositories storing human data (even deidentified) include (1) fidelity to consent, (2) restricted use compliant, (3) privacy, (4) plan for breach, (5) download control, (6) violations, and (7) data request review.
